# Kidney Outcomes in Patients With Advanced Heart Failure Treated With Ventricular Assist Devices

**DOI:** 10.1016/j.xkme.2025.101027

**Published:** 2025-05-16

**Authors:** Lyle W. Baker, Tambi Jarmi, Michael A. Mao, Ivan E. Porter, Christopher L. Trautman, Parag C. Patel, Yaohua Ma, David O. Hodge, Nabeel Aslam

**Affiliations:** 1 Division of Nephrology and Hypertension, Mayo Clinic, Jacksonville, FL; 2 Division of Kidney and Pancreas Transplant, Mayo Clinic, Jacksonville, FL; 3 Division of Heart Failure and Transplant, Mayo Clinic, Jacksonville, FL; 4 Division of Quantitative Health Sciences, Mayo Clinic, Jacksonville, FL

**Keywords:** Ventricular assist device, acute kidney injury, chronic kidney disease, advanced-stage heart failure, mechanical circulatory support, kidney replacement therapy, acute renal failure, renal dysfunction, chronic renal failure, congestive heart failure, dialysis, renal replacement therapy

## Abstract

**Rationale & Objective:**

Ventricular assist devices (VADs) are used for advanced heart failure, but their impact on kidney function remains unclear. This study evaluated changes in kidney function following VAD implantation, including acute kidney injury (AKI) incidence and need for kidney replacement therapy (KRT).

**Study Design:**

A retrospective cohort study analyzing longitudinal kidney function outcomes post-VAD placement.

**Setting & Participants:**

Adult patients who underwent durable VAD placement (2009-2019) at a single center were included. Patients were stratified into chronic kidney disease (CKD) (estimated glomerular filtration rate [eGFR] <60 mL/min/1.73m^2^) and non-CKD (eGFR ≥60 mL/min/1.73m^2^) groups.

**Exposures & Predictors:**

The VAD implantation was the primary intervention, with baseline kidney function modifying its impact on post-VAD kidney function.

**Outcomes:**

Primary outcomes were changes in eGFR and creatinine at 3-months and 12-months post-VAD. Secondary outcomes included AKI incidence, KRT requirement, and postdischarge AKI within 1 year.

**Analytical Approach:**

Descriptive statistics and comparative analyses, including Wilcoxon rank sum, χ^2^, and paired *t* tests, were used to assess differences. Significance was set at *P* < 0.05.

**Results:**

Among 160 patients (82% male and 69% White), patients with CKD were older with a higher prevalence of diabetes, vasodilator use, and inotrope use. At 3 months, kidney function improved in patients with CKD (eGFR +17, *P* < 0.001) but declined by 12 months (eGFR +7, *P* = 0.03). The non-CKD group had a smaller improvement at 3 months (eGFR +8, *P* = 0.004) that was not sustained. AKI requiring KRT occurred in 14%, with 45% in-hospital mortality; and 41% discontinued KRT before discharge. Post-VAD AKI occurred in 21%. Half of the patients underwent heart transplant, which was associated with worsening kidney function at 1-year.

**Limitations:**

Single-center design limits generalizability.

**Conclusions:**

The VAD placement initially improves kidney function, particularly in CKD patients, but this effect diminishes over time. AKI and KRT use are common, highlighting the need for close kidney monitoring post-VAD.

## Plain-Language Summary

Ventricular assist devices (VADs) help patients with advanced heart failure by supporting heart function, but their impact on kidney health is not well understood. Because kidney disease is common in heart failure and linked to worse outcomes, we studied how kidney function changes after VAD placement. We compared patients with and without chronic kidney disease and found that kidney function improved in the first 3 months, especially in those with chronic kidney disease. However, this benefit declined over the first year. Some patients developed acute kidney injury requiring dialysis, which significantly increased their risk of death. These findings highlight the importance of closely monitoring kidney health in VAD patients to improve long-term outcomes.

Durable ventricular assist devices (VADs) are increasingly being used for the treatment of advanced-stage heart failure. However, the long-term effects of VADs on kidney function is still largely under investigation. More than 6 million American adults experience chronic heart failure (CHF).^1^ Cardiorenal syndrome (CRS) types I and II are common causes of kidney impairment in patients with CHF, with CRS type I prevalence ranging between 25%-50% in this patient population.^2^^,^^3^ Chronic kidney disease (CKD) from CRS is common, with an estimated two-thirds of patients with CHF having CKD stage III or worse.^4^^,^^5^ Among patients with CHF, those with CKD have a 2 to 3 time higher mortality risk than in those without CKD.^4^^,^^6^ Over the last few decades, the development of VADs has offered patients with advanced-stage heart failure a means of circulatory support either as destination therapy or as a bridge to heart transplant. The VADs differ in ventricle type (left, right, or biventricular) and pump flow (pulsatile or continuous-flow). Continuous-flow devices have largely replaced pulsatile devices because of decreased rates of device failure, infection, thrombosis, bleeding, right heart failure, and kidney failure.^7^^,^^8^ However, these complications still exist with modern continuous-flow VADs.^9^ Regardless, VADs have proven benefits over optimal medical therapy in reducing all-cause mortality and improving quality of life in patients with advanced heart failure.^10^ However, studies looking at kidney outcomes of VADs remains limited and inconclusive.

A few studies have shown either stable or initial improvement in baseline kidney function immediately after VAD placement.^7^^,^^11^^,^^12^ The development of acute kidney injury (AKI) after VAD placement is also a recognized complication.^7^ Further research is needed to understand the incidence and causes of AKI and CKD after VAD placement. Therefore, we designed this single-center, retrospective cohort study exploring how VADs affect kidney outcomes regarding acute and CKD.

## Methods

### Study Design

This single-center, retrospective cohort study was conducted at the Mayo Clinic in Jacksonville, Florida, United States, using a registry of patients who underwent VAD placement at the Mayo Clinic in Florida. The institutional review board reviewed and approved the protocol. All patients received post-VAD care at the same institution until discharge. After discharge, patients had outpatient follow-ups with laboratory monitoring over the course of 1-year. All adult patients (aged ≥18 years) with advanced heart failure who underwent VAD placement from January 1, 2009, to December 31, 2019, were included in this cohort. Kidney function was determined using the 2009 CKD epidemiology collaboration (CKD-EPI) equation to calculate the estimated glomerular filtration rate (eGFR). The CKD staging was determined using the 2012 Kidney Disease Improving Global Outcomes (KDIGO) CKD guidelines based on GFR.^13^ Patient were categorized into 2 cohorts based on pre-VAD kidney function using a GFR cutoff of 60 mL/min/1.73m^2^, in accordance with KDIGO guidelines, which define CKD as GFR <60 mL/min/1.73m^2^ persisting for at least 3 months. This threshold is a widely accepted standard for distinguishing patients with moderate-to-severe kidney dysfunction from those with preserved kidney function.

### Data Collection

Data including patient demographics (age at the time of VAD placement, sex, race, and ethnicity), comorbid conditions (ischemic cardiomyopathy, nonischemic cardiomyopathy, diabetes mellitus type 1 or 2, essential hypertension, pulmonary hypertension, CKD, active or prior tobacco use, obesity, peripheral vascular disease, and depression), medications, and laboratory data were inputted using the research electronic data capture software by the primary author. The coauthors reviewed the data for accuracy. All comorbid conditions were determined based on a documented diagnosis in the patient’s chart except CKD, which was determined through a manual chart review.             

Baseline kidney function was determined through a manual chart review assessing eGFR values over the 3 months preceding VAD placement. Patients were classified into a CKD cohort if they had an eGFR <60 mL/min/1.73m^2^ persisting for at least 3 months before VAD placement, consistent with KDIGO criteria for CKD. This approach distinguished patients with true CKD from those experiencing a transient decline in kidney function because of acute kidney disease/injury at the time of VAD placement.

Data were collected on patient use of any of the following medications within 60 days before VAD placement: β-blockers, angiotensin-converting enzyme inhibitors, angiotensin II receptor blockers, calcium channel blockers, vasodilators (hydralazine, isosorbide, nitroglycerin, or minoxidil), α-blockers, diuretics, positive inotropic agents (dopamine, dobutamine, epinephrine, norepinephrine, angiotensin II, or milrinone), and psychiatric medications. The laboratory data, including plasma creatinine, eGFR based on CKD-EPI equation, urine protein-creatinine ratio, and plasma albumin, were recorded pre-VAD as a baseline. These same laboratory values were recorded 24 hours post-VAD, 48 hours post-VAD, 1-month post-VAD, at time of hospital discharge post-VAD, 3 months post-VAD ± 30 days, 6 months post-VAD ± 30 days, 9 months post-VAD ± 30 days, and 12 months post-VAD ± 30 days.

These same data were also collected for patients who underwent heart transplant after VAD placement using the same time intervals over 1 year. The need for kidney replacement therapy (KRT) post-VAD and any additional post-VAD hospitalizations with associated AKI at the Mayo Clinic Florida were recorded for 1-year post-VAD outcomes. AKI was defined using the KDIGO classification based on changes in serum creatinine level (increase by ≥0.3 mg/dL within 48 hours or an increase in creatinine ≥1.5 times baseline within 7 days) or initiation of KRT.^14^ All patients were followed until either 1 year post-VAD, 1-year postheart transplant if the transplant occurred within 1 year post-VAD, or until death within 1 year after VAD or heart transplant.

### Inclusion and Exclusion Criteria

All adult patients aged 18 years and above with advanced heart failure who underwent VAD placement at the Mayo Clinic in Florida from 2009 to 2019 were included in this study. Patients with a diagnosis of kidney failure receiving KRT before VAD placement were excluded from this study.

### Statistical Methods

This study summarized numerical variables using the median (range) and mean (standard deviation), whereas categorical variables were summarized with frequency and percentage. The Wilcoxon rank sum test was used to evaluate differences for numerical variables, and the Pearson χ^2^ test was used to assess the proportional differences for discrete variables between 2 cohorts of patients: non-CKD patients and patients with CKD. Paired *t* test was used to compare changes in plasma creatinine and eGFR at pre-VAD values to varying post-VAD values over time. All tests were 2-sided and *P* values of <0.05 were considered statistically significant.

## Results

Total of 160 patients received a VAD from 2009 to 2019 with a median age of 57 years, 82% men, and 69% White. The cohort was divided into 2 groups: non-CKD pre-VAD (n = 99) and CKD pre-VAD (n = 61). Patients in the CKD group were older at the time of VAD implant (61 vs 55 years; *P* = 0.02), had a higher percentage of men (93% vs 75%; *P* = 0.003), and more likely to be on a vasodilator (39% vs 20%; *P* = 0.008). There was a trend toward a higher prevalence of diabetes mellitus (54% vs 38%; *P* = 0.05) and inotropic agents (93% vs 83%; *P* = 0.05). Of those with CKD (n = 61), 51% had stage III-A, 41% had stage III-B, 7% had stage IV, and 1% had stage V without dialysis. See Table 1.

**Table 1:** Patient Demographics and Baseline Characteristics

	Non-CKD Pre-VAD n = 99	CKD Pre-VAD n = 61	Total n = 160	*P*
**Age at time of VAD placement, y**				0.02
Median (range)	55 (19-74)	61 (22-73)	57 (19-74)	
Mean ± SD	54 ± 13.8	59 ± 9.4	56 ± 12.6	
Males, % (n)	75% (74)	93% (57)	82% (131)	0.003
Race, % (n)				0.91
White	70% (69)	67% (41)	69% (110)	
African American	24% (24)	28% (17)	26% (41)	
Asian	3% (3)	2% (1)	2% (4)	
Unknown	3% (3)	3% (2)	3% (5)	
Hispanic or Latino Ethnicity, % (n)	7% (7)	3% (2)	6% (9)	0.43
Comorbid conditions, % (n)				
Ischemic cardiomyopathy	48% (48)	44% (27)	47% (75)	0.60
Nonischemic cardiomyopathy	52% (51)	56% (34)	53% (85)	
Diabetes mellitus Type 1 or 2	38% (38)	54% (33)	44% (71)	0.05
Home insulin use	61% (23)	66% (22)	63% (45)	0.59
Essential hypertension	55% (54)	59% (36)	56% (90)	0.58
Pulmonary hypertension	23% (23)	33% (20)	27% (43)	0.19
Active or previous tobacco use	57% (56)	61% (37)	58% (93)	0.61
Obesity (BMI >30 kg/m^2^)	41% (41)	44% (27)	43% (68)	
Peripheral vascular disease	6% (6)	5% (3)	6% (9)	0.76
Depression	27% (27)	26% (16)	27% (43)	0.89
Medications, % (n)				
β-Blockers	77% (77)	75% (46)	77% (123)	0.73
ACE-inhibitors	52% (51)	34% (21)	45% (72)	0.04
Angiotensin II receptor blockers	21% (21)	26% (16)	23% (37)	0.47
ACE-I or ARB	73% (72)	61% (37)	68% (109)	0.11
Calcium channel blockers	2% (2)	0% (0)	1% (2)	0.26
Vasodilators	20% (20)	39% (24)	28% (44)	0.008
α-Blockers	4% (4)	3% (2)	4% (6)	0.81
Diuretics	98% (97)	98% (60)	98% (157)	0.86
Positive inotropic agents	83% (82)	93% (57)	87% (139)	0.05
Psychiatric medications	38% (38)	31% (19)	36% (57)	0.35
Laboratory reports pre-VAD, median (mean)				
Plasma creatinine, mg/dL	1.1 (1.2)	2.0 (2.2)	1.4 (1.6)	
EGFR, mL/min/1.73m^2^	73 (71)	36 (38)	57 (59)	
Plasma albumin, g/dL	3.7 (3.7)	3.8 (3.8)	3.8 (3.7)	
Plasma hemoglobin, g/dL	10.9 (11.1)	10.6 (10.8)	10.9 (11.0)	
Ejection fraction, median (mean)	16 (17)	16 (17)	16 (17)	
Initial VAD Type, % (n)				
HeartMate II	55% (54)	57% (35)	56% (89)	
HeartWare	38% (38)	38% (23)	38% (61)	
HeartMate III	3% (3)	3% (2)	3% (5)	
Thoratec	2% (2)	2% (1)	2% (3)	
HeartMate XVE	1% (1)	0% (0)	0.6% (1)	
CentriMag	1% (1)	0% (0)	0.6% (1)	

Abbreviations: ACE, angiotensin-converting enzyme; ARB, angiotensin-receptor blocker; BMI, basic metabolic index; CKD, chronic kidney disease; EGFR, epidermal growth factor receptor; VAD, ventricular assist device.

A total of 94% of the VADs were continuous and almost all (98%) were left-sided ventricular assist devices. The distribution of VAD types used between the 2 cohorts were essentially the same with 56% being HeartMate II, 38% being HeartWare, 3% being HeartMate III, 2% being Thoratec, 0.6% being HeartMate XVE, and 0.6% being CentriMag. See Table 1. 9% of patients required a subsequent VAD exchange after their original placement.

### Change in Plasma Creatinine and eGFR Over Time Post-VAD

**Figure 1:**
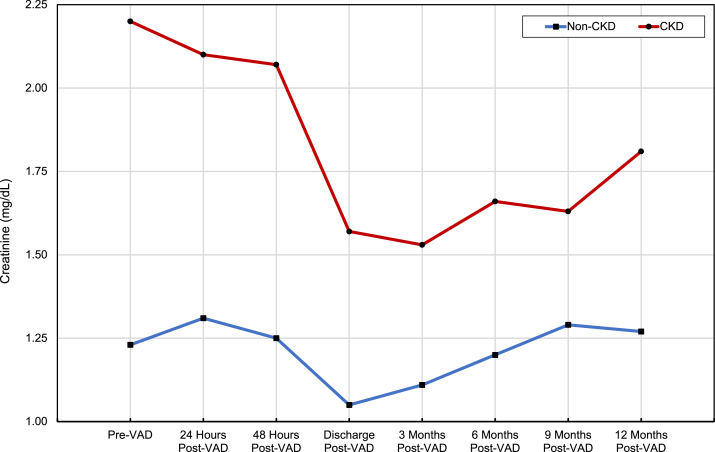
Change in plasma creatinine over time post-VAD. Red line: CKD patients; Blue line: non-CKD patients. CKD, chronic kidney disease.

**Figure 2:**
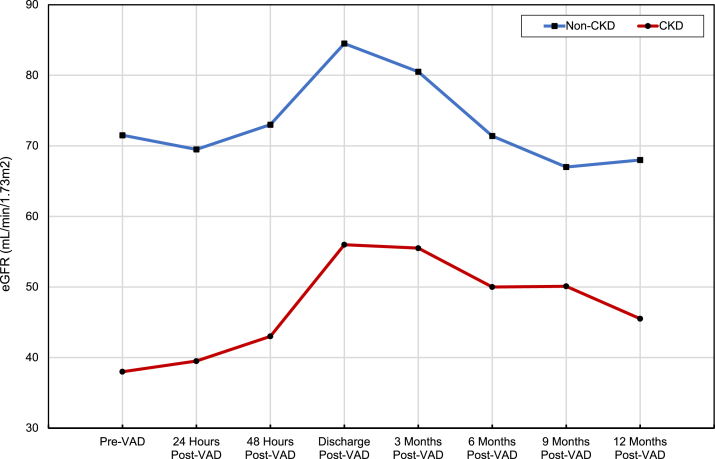
Change in plasma eGFR over time post-VAD. Red line: CKD patients; Blue line: non-CKD patients. eGFR, estimated glomerular filtration rate; CKD, chronic kidney disease.

There was a statistically significant improvement in plasma creatinine (−0.53 mg/dL; 95% CI [−0.68 to −0.38]; *P* ≤ 0.001) and eGFR (+17 mL/min/1.73m^2^; 95% CI [12-22]; *P* ≤ 0.001) from pre-VAD value to 3-month post-VAD value in the CKD group. At 12 months, the improvement in plasma creatinine (−0.25 mg/dL; 95% CI [−0.47 to −0.02]; *P* = 0.03) and eGFR (+7 mL/min/1.73m^2^; 95% CI [0.66-12.84]; *P* = 0.03) persisted to a lesser degree in the CKD group. These changes in plasma creatinine and eGFR suggest that kidney function in patients with CKD improves following VAD placement; however, this improvement declines over the first year after VAD placement. In comparison, among the non-CKD group, there was a statistically significant increase in eGFR (+8 mL/min/1.73m^2^; 95% CI [3-14]; *P* = 0.004) from pre-VAD value to 3-month post-VAD, although no significant difference in creatinine (−0.10 mg/dL; 95% CI [−0.22 to 0.02]; *P* = 0.09). In the non-CKD group, there was no significant difference in creatinine (+0.06 mg/dL; 95% CI [−0.08 to 0.20]; *P* = 0.40) or eGFR (−3.19 mL/min/1.73m^2^; 95% CI [−10.07 to 3.69]; *P* = 0.36) when comparing eGFR from pre-VAD value with 12-month post-VAD value. The change in the plasma creatinine and eGFR during the first-year post-VAD placement is summarized in Figs 1 and 2; Table 2.

**Table 2:** Statistical Differences of Creatinine and eGFR Over Time Post-VAD

	Comparison Groups	Estimated Difference (95% CI)	*P*	Result
Comparing creatinine in CKDcohort (mg/dL)	3 Mo post-VAD vs pre-VAD	−0.53 (−0.68 to −0.38)	< 0.001	Creatinine significantly less than pre-VAD.
	12 Mo post-VAD vs pre-VAD	−0.25 (−0.47 to −0.02)	0.03	Creatinine significantly less than pre-VAD.
Comparing eGFR in CKD cohort (mL/min/1.73m^2^)	3 Mo post-VAD vs pre-VAD	17 (11.88-22.12)	< 0.001	EGFR significantly more than pre-VAD.
	12 Mo post-VAD vs pre-VAD	6.75 (0.66-12.84)	0.03	EGFR significantly more than pre-VAD.
Comparing creatinine in non-CKD cohort (mg/dL)	3 Mo post-VAD vs pre-VAD	−0.10 (−0.22-0.02)	0.09	No statistically significant difference.
	12 Mo post-VAD vs pre-VAD	0.06 (−0.08 to 0.20)	0.40	No statistically significant difference.
Comparing eGFR in non-CKD cohort (mL/min/1.73m^2^)	3 Mo post-VAD vs pre-VAD	8.19 (2.61-13.77)	0.004	EGFR significantly more than pre-VAD.
	12 Mo post-VAD vs pre-VAD	−3.19 (−10.07 to 3.69)	0.36	No statistically significant difference.

*Note:* Paired *t* test was used to compare changes in plasma creatinine and eGFR at pre-VAD values to different post-VAD values over time.

Abbreviations: CKD, chronic kidney disease; eGFR, estimated glomerular filtration rate; VAD, ventricular assist device.

### Length of Hospital Stay and 1-Year Mortality Post-VAD

There was no significant difference in median length of hospital stay following VAD placement among non-CKD and CKD groups (19 vs 21 days; *P* = ns). There was no significant difference in 1-year mortality post-VAD placement among non-CKD and CKD groups (16% vs 13%; *P* = ns).

### KRT During Hospitalization for VAD Placement

Of the 160 VAD patients, 22 patients (14%) developed AKI that required initiation of KRT during their hospitalization for VAD placement. There was no statistically significant difference in the percentage of patients that required KRT between the CKD and non-CKD groups (16% vs 12%; *P* = 0.45). Among the CKD group, those who required KRT after VAD placement had a statistically significant lower pre-VAD eGFR compared with those who did not (mean pre-VAD eGFR 25 vs 40 mL/min/1.73m^2^; *P* ≤ 0.001). See Table 3.

**Table 3:** Comparing eGFR Pre-VAD in Patients that Required KRT Post-VAD

	Required KRT n = 22	Did Not Require KRT n = 138	Total n = 160	*P*
**CKD pre-VAD**				
Number of patients	10	51	61	< 0.001
eGFR, median (range)	27 (5-36)	38 (24-79)	36 (5-79)	
eGFR, mean ± SD	25.3 ± 8.6	40 ± 11.9	37.9 ± 12.7	
**Non-CKD pre-VAD**				
Number of patients	12	87	99	0.12
eGFR, median (range)	65 (20-98)	73 (24-146)	73 (20-146)	
eGFR, mean ± SD	60 ± 22.5	73 ± 22.3	72 ± 22.6	

Abbreviations: eGFR, estimated glomerular filtration rate; KRT, kidney replacement therapy; VAD, ventricular assist device.

Of the 22 patients that required KRT, in-hospital mortality during VAD placement was 45% (10 patients). 41% of patients (9 of 22) that required initiation of KRT had kidney function recovery that allowed KRT discontinuation before discharge. There was no statistically significant difference in the percentage of patients who had kidney function recovery and could discontinue KRT between the CKD and non-CKD groups (44% vs 56%). All 3 patients that were discharged on KRT after hospitalization for VAD placement died within 1.5 years after VAD placement.

### Hospitalizations Following VAD Placement With Associated AKI

All patients readmitted to the hospital following VAD placement were assessed for AKI during each of their subsequent hospitalizations for up to 1 year. 60% of patients had at least one hospitalization within 1 year post-VAD placement (62% in non-CKD group vs 57% in CKD group; *P* = 0.60). The incidence of AKI during any hospitalization within 1-year post-VAD placement was 21% (18% in non-CKD group vs 25% in CKD group; *P* = 0.33). Three patients in CKD group had > 2 episodes of AKI during the hospitalization within 1 year post-VAD placement. Five patients had AKI requiring initiation of KRT during a subsequent hospitalization within 1-year post-VAD placement. Overall, there was no significant difference in the incidence of hospitalization and development of AKI between the non-CKD and CKD groups. See Table 4.

**Table 4:** Hospitalizations After VAD Placement with Associated AKI

	Non-CKD Pre-VAD n = 99	CKD Pre-VAD n = 61	Total n = 160	*P*
Hospitalized within 1-y post-VAD, % (n)	62% (61)	57% (35)	60% (96)	0.56
AKI in 1-y post-VAD during any hospitalization, % (n)	18% (18)	25% (15)	21% (33)	0.33
**Number of AKIs**				
0	82% (81)	75% (46)	79% (127)	0.15
1	13% (13)	18% (11)	15% (24)	
2	5% (5)	2% (1)	4% (6)	
3	0% (0)	3% (2)	1% (2)	
4	0% (0)	2% (1)	1% (1)	
≥5	0% (0)	0% (0)	0% (0)	
AKI Requiring KRT	(3%) 3	(3%) 2	(3%) 5	NS

Abbreviations: AKI, acute kidney injury, CKD, chronic kidney disease; KRT, kidney replacement therapy; VAD, ventricular assist device.

### Change in Plasma Creatinine and eGFR Over Time Postheart Transplant

**Figure 3:**
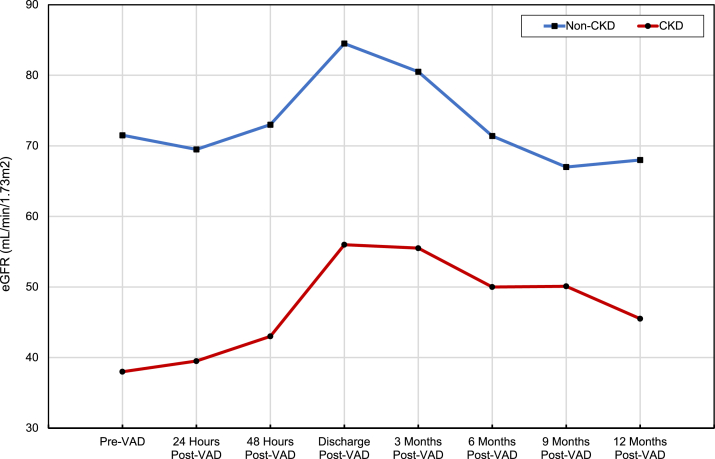
Change in plasma creatinine over time postheart transplant. Red line: CKD patients; Blue line: non-CKD patients. CKD, chronic kidney disease.

**Figure 4:**
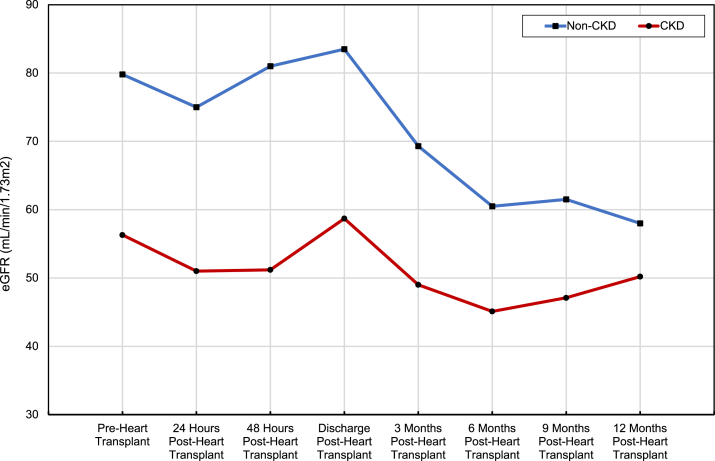
Change in plasma eGFR over time postheart transplant. Red line: CKD patients; Blue line: non-CKD patients. CKD, chronic kidney disease.

A total of 81 patients received a heart transplant after VAD (50 patients received heart transplants within 1 year post-VAD, whereas 31 patients received heart transplants >1 year post-VAD). See Table 5. Heart transplant recipients were younger (56 vs 62 years; *P* = 0.002) and had a lower prevalence of depression (18.5% vs 35%; *P* = 0.02) in comparison with nonrecipients. Forty-seven out of 99 (47%) patients from the non-CKD group received heart transplants compared with 34 out of 61 (56%) patients from the CKD group. Three of the 34 patients in the CKD group underwent simultaneous heart-kidney transplant and were thus excluded when analyzing changes in creatinine and eGFR after heart transplant. Plasma creatinine and eGFR values were recorded at 3 months, 6 months, 9 months, and 12 months postheart transplant. The change in the plasma creatinine and eGFR during the first-year postheart transplant compared with pretransplant is summarized in Figs 3 and 4; Table 6. At 3 months postheart transplant, there was a statistically significant decrease in eGFR in both the CKD group (−8.14 mL/min/1.73m^2^; 95% CI [−15.68 to −0.61]; *P* = 0.04) and non-CKD group (−11.07 mL/min/1.73m^2^; 95% CI [−17.97 to −4.17]; *P* = 0.002). At 12 months postheart transplant, this decline in eGFR was sustained in the CKD group (−8.58 mL/min/1.73m^2^; 95% CI [−17.1 to −0.05]; *P* = 0.05) and further decreased in the non-CKD group (−19.74 mL/min/1.73m^2^; 95% CI [−26.58 to −12.89]; *P* ≤ 0.001).

**Table 5:** Transplant and Mortality Post-VAD

	Non-CKD Pre-VAD n = 99	CKD Pre-VAD n = 61	Total n = 160	*P*
Heart transplant within 1-y post-VAD placement, % (n)	29% (29)	34% (21)	31% (50)	0.50
Kidney transplant within 1-y post-VAD placement, % (n)	0% (0)	5% (3)^a^	2% (3)	0.03
Death within 1-y post-VAD placement, % (n)	16% (16)	13% (8)	15% (24)	0.60

Abbreviations: CKD, chronic kidney disease; VAD, ventricular assist device.

a Underwent simultaneous heart-kidney transplant.

**Table 6:** Statistical Differences of Creatinine and eGFR Over Time Postheart Transplant

	Comparison Groups	Estimated Difference(95% CI)	*P*	Result
Comparing creatinine in CKDcohort (mg/dL)	3 Mo postheart transplant vs preheart transplant	0.46 (−0.11 to 1.03)	0.11	No statistically significant difference.
	12 Mo postheart transplant vs preheart transplant	0.26 (0.02-0.49)	0.03	Creatinine significantly higher than preheart transplant.
Comparing eGFR in CKDcohort (mL/min/1.73m^2^)	3 Mo postheart transplant vs preheart transplant	−8.14 (−15.68 to −0.61)	0.04	EGFR significantly less than preheart transplant.
	12 Mo postheart transplant vs preheart transplant	−8.58 (−17.1 to −0.05)	0.05	EGFR significantly less than preheart transplant.
Comparing creatinine innon-CKDcohort (mg/dL)	3 Mo postheart transplant vs preheart transplant	0.19 (0.08-0.29)	0.001	Creatinine significantly higher than preheart transplant.
	12 Mo postheart transplant vs pre-heart transplant	0.36 (0.24-0.47)	< 0.001	Creatinine significantly higher than preheart transplant.
Comparing eGFR in non-CKDcohort (mL/min/1.73m^2^)	3 Mo post-heart transplant vs preheart transplant	−11.07 (−17.97 to −4.17)	0.002	EGFR significantly less than pre-heart transplant.
	12 mos postheart transplant vs preheart transplant	−19.74 (−26.58 to −12.89)	< 0.001	EGFR significantly less than preheart transplant.

*Note:* Paired T-test was used to compare changes in plasma creatinine and eGFR at preheart transplant values to different postheart transplant values over time.

Abbreviations: CKD, chronic kidney disease; EGFR, epidermal growth factor receptor; eGFR, estimated glomerular filtration rate.

## Discussion

This study examined the short-term and long-term effects of VAD implantation on kidney function between CKD and non-CKD patient populations. Results indicated an improvement in eGFR at 3 months after VAD placement in CKD (+17 mL/min/1.73m^2^) and non-CKD patients (+8 mL/min/1.73m^2^). However, the magnitude of this improvement 1-year after VAD placement diminished in CKD patients (remaining only 7 mL/min/1.73m^2^ higher as compared with pre-VAD value) and was nonsustained in non-CKD patients. The presence of CKD was not associated with longer length of hospital stay after VAD placement (21 vs 19 days; *P* = ns), need for KRT (16% vs 12%; *P* = 0.45), development of AKI (25% vs 18%; *P* = 0.33), or death (13% vs 16%; *P* = 0.60) over 1-year follow-up.

Previous studies have reported improvements in eGFR up to 6 months post-VAD placement and subsequently a gradual decline in eGFR during the 6-month follow-up.^15^^,^^16^ Our study confirms these findings and extends the follow-up period to 1-year. Brisco et al^17^ reported in their cohort an improvement in eGFR of only 2.6 mL/min/1.73m^2^ over the pre-VAD value at 1 year of follow-up. In contrast, in our cohort, the CKD group had an improvement in eGFR of 7 mL/min/1.73m^2^ over the pre-VAD value at 1-year follow-up, whereas the non-CKD group did not have a sustained improvement in eGFR. We hypothesize that this 1-year difference in eGFR between CKD and non-CKD groups is related to inadequate renal perfusion from CRS type II in the CKD group that improved after VAD placement and resulted in a higher initial increase in eGFR in comparison with the non-CKD group (17 mL/min/1.73m^2^ vs 8 mL/min/1.73m^2^, respectively). Both groups had a similar subsequent decline in eGFR from 3 months post-VAD to 12 months post-VAD (Δ eGFR −10 mL/min/1.73m^2^ in the CKD group (17 mL/min/1.73m^2^ at 3 months to 7 mL/min/1.73m^2^ at 12 months] vs −11 mL/min/1.73m^2^ in the non-CKD group [8 mL/min/1.73m^2^ at 3 months to −3 mL/min/1.73m^2^ at 12 months). Possible explanations for progressive decline in eGFR over the 1-year follow-up include pigment nephropathy from subclinical chronic hemolysis, lack of pulsatile flow resulting in increased activation of renin-angiotensin-aldosterone system pathway and small-vessel renovascular disease, development of right ventricular dysfunction causing renal congestion, and addition of new medications that can affect renal hemodynamics.^18^ Further studies are needed to explore these differences.

Previous studies have reported incidences and outcomes of AKI immediately post-VAD placement. In a retrospective cohort of 241 patients with VAD, 70% developed AKI (stages I, II, or III) within the first 7 days post-VAD placement, with stages II and III AKI being associated with higher 1-year mortality risk (HR, 2.2).^19^ Harmon et al^20^ reported that 28% of patients developed stages II or III AKI in their retrospective cohort of 246 patients who underwent VAD placement during initial VAD hospitalization. In our cohort, 14% of patients developed stage III AKI requiring KRT during initial VAD hospitalization. There is a paucity of data on the incidence of AKI and the impact of CKD on the development of AKI after VAD placement. Our study fills this gap and provides insight into episodes of AKI that develop during subsequent hospitalizations over the first year after discharge from initial VAD placement. In our cohort, 60% of patients had at least one hospitalization within the first year after VAD placement. The incidence of AKI of any stage was 21% in these subsequent hospitalizations over the first year, with no statistical difference in incidence between non-CKD and CKD groups (18% vs 25%; *P* = 0.33). Our study advances understanding of the incidence of AKI during the first year post-VAD placement.

In our cohort, there was no significant difference in the percentage of patients who received heart transplants within 1 year post-VAD when comparing CKD and non-CKD patient populations (34% vs 29%; *P* = 0.50). Singh et al^21^ previously compared changes in creatinine clearance (CrCl) in patients with VAD who underwent heart transplant. They found that patients overall had a statistically significant decrease in CrCl by 20 mL/min one year after heart transplant. In addition, they found that patients with a pre-VAD CrCl >65 had a greater decrease in CrCl from pretransplant to 1 year post-transplant (−24 mL/min) in comparison with patients with a pre-VAD CrCl ≤65 (−16 to −17.5 mL/min).^21^ Our study also confirmed these findings. We found a statistically significant decrease in eGFR one year after heart transplant that was also greater in the non-CKD group (−20 mL/min/1.73m^2^; *P* ≤ 0.001) than in the CKD group (−9 mL/min/1.73m^2^; *P* = 0.05). We hypothesize that this decline in eGFR after heart transplantation is primarily related to calcineurin inhibitor nephrotoxicity. Further studies are needed to understand this differential change in eGFR between non-CKD and CKD patients after heart transplant.

The strengths of our study include detailed data about the eGFR change over the first year post-VAD placement in CKD and non-CKD patients, the outcome of AKI patients requiring KRT immediately after VAD placement, and evaluation for the incidence of AKI during subsequent hospitalizations. In addition, this study provides detailed 1-year follow-up data on eGFR changes among patients with VAD who receive heart transplants.

The limitation of this study includes being a single center, retrospective review with its potential for selection bias. However, we included all consecutive patients who had VAD placement over the study period. The change in clinical practice over the 10 years can affect the outcome; however, patients in both groups were of similar distribution for the time. Although survival bias is a potential concern, the similar 1-year post-VAD mortality between the CKD and non-CKD groups (13% vs 16%; *P* = 0.60) suggests that differential survival is unlikely to have significantly impacted the observed eGFR trends between the 2 cohorts. Nonetheless, patients with severe post-VAD kidney dysfunction may have had higher mortality, which could still influence later kidney function assessments. Our cohort included predominantly White males and thus may not necessarily be reflective of outcomes in females and other races. However, the demographics of our cohort is similar to other cohorts in this field of study and the INTERMACS registry from 2012-2015, with similar proportion of men, whites, and causes of heart failure.^9^^,^^15^^,^^22^, ^23^, ^24^ This merits further studies to explore the reasons for sex and racial disparities in receiving advance heart failure therapies. The 2009 CKD-EPI equation, which includes a race coefficient, was used at the time of data collection in 2020 because it was still considered the standard for estimating GFR at that time. Plasma creatinine was used in our study to estimate changes in GFR over time. Recognizing that patients may have changes in muscle mass after VAD placement or heart transplant, which could affect creatinine values, future studies using other biomarkers of kidney function such as cystatin C should be considered. Although proteinuria/albuminuria is an important biomarker for chronic kidney disease, because of a limited number of patients having pre-VAD random urine assessments for proteinuria, this biomarker was excluded in our differentiation between the CKD and non-CKD cohorts.

## Conclusion

VADs are associated with initial improvement in kidney function at 3 months after VAD insertion in both CKD and non-CKD patients with advanced heart failure. The degree of this improvement is more pronounced in patients with CKD. However, the magnitude of this improvement 1 year after VAD placement diminishes in CKD patients and is not sustained in non-CKD patients. The AKI during hospitalization for VAD placement is frequent with nearly 15% of patients requiring initiation of KRT, which is associated with significant in-hospitality mortality (45%). Less than half of patients who require KRT have adequate recovery to allow KRT discontinuation before discharge. The AKI within 1 year of VAD placement is also common, with just >20% of patients having a subsequent hospitalization with AKI of any stage.
